# Enhancing thromboresistance of neurovascular nickel-titanium devices with responsive heparin hydrogel coatings

**DOI:** 10.1136/jnis-2024-021836

**Published:** 2024-05-17

**Authors:** Manfred F Maitz, Daniel P O Kaiser, Ani Cuberi, Rafaela Weich Hernández, Ruben Mühl-Benninghaus, Toshiki Tomori, Matthias Gawlitza

**Affiliations:** 1 Max Bergmann Center of Biomaterials, Leibniz Institute of Polymer Research Dresden, Dresden, Sachsen, Germany; 2 Institute of Neuroradiology, University Hospital Carl Gustav Carus, Technische Universität Dresden, Dresden, Sachsen, Germany; 3 Institute of Radiology, University Hospital Carl Gustav Carus, Technische Universität Dresden, Dresden, Germany; 4 Institute of Radiology, Städtisches Klinikum Lüneburg, Lüneburg, Germany; 5 Department of Diagnostic and Interventional Neuroradiology, University Medical School of Saarland, Homburg/Saar, Germany; 6 Institute of Neuroradiology, University Hospital Leipzig, Leipzig, Sachsen, Germany

**Keywords:** Stent, Pharmacology, Material

## Abstract

**Background:**

Neurointerventional devices, particularly laser-cut thin-strut stents made of self-expanding nickel-titanium alloy, are increasingly utilized for endovascular applications in intracranial arteries and dural venous sinuses. Preventing thrombosis and stroke necessitates systemic anticoagulant and antiplatelet therapies with the risk of bleeding complications. Antithrombotic coatings present a promising solution.

**Methods:**

In this study, we investigated the potential of hydrogels composed of four-armed poly(ethylene glycol) (starPEG) and heparin, with or without coagulation-responsive heparin release, as coatings for neurovascular devices to mitigate blood clot formation. We evaluated the feasibility and efficacy of these coatings on neurovascular devices through in vitro Chandler-Loop assays and implantation experiments in the supra-aortic arteries of rabbits.

**Results:**

Stable and coagulation-responsive starPEG-heparin hydrogel coatings exhibited antithrombotic efficacy in vitro, although with a slightly reduced thromboprotection observed in vivo. Furthermore, the hydrogel coatings demonstrated robustness against shear forces encountered during deployment and elicited only marginal humoral and cellular inflammatory responses compared with the reference standards.

**Conclusion:**

Heparin hydrogel coatings offer promising benefits for enhancing the hemocompatibility of neurointerventional devices made of self-expanding nickel-titanium alloy. The variance in performance between in vitro and in vivo settings may be attributed to differences in low- and high-shear blood flow conditions inherent to these models. These models may represent the differences in venous and arterial systems. Further optimization is warranted to tailor the hydrogel coatings for improved efficacy in arterial applications.

**WHAT IS ALREADY KNOWN ON THIS TOPIC**

- Antithrombotic coatings are of great interest in enhancing the hemocompatibility of neurointerventional devices.

**WHAT THIS STUDY ADDS**

- This study demonstrates the potential of hydrogels composed of four-armed poly(ethylene glycol) and heparin as coatings for neurovascular devices, showing efficacy in mitigating blood clot formation both in vitro and in vivo.

**HOW THIS STUDY MIGHT AFFECT RESEARCH, PRACTICE, OR POLICY**

- These findings suggest a new coating technology for the armamentarium to enhance the hemocompatibility of neurointerventional devices.

## Introduction

 Endovascular procedures pose a significant risk of thromboembolic complications due to the potential clot formation triggered by the exposure of bare-metal wires and catheters to the patient’s circulation. Thromboembolic complications represent critical risks in neurointerventions, potentially leading to ischemic brain damage and stroke, causing severe morbidity or disability.^1 2^ Consequently, the intervention frequently requires preventive anticoagulant and antiplatelet regimens, which are inherently associated with an increased risk of bleeding. In a meta-analysis of patients with acutely ruptured intracranial aneurysms undergoing endovascular treatment, the rate of ventriculostomy-related hemorrhage was 20.9% among patients receiving antiplatelet therapy, compared with 9% in the control group not receiving antiplatelet therapy (p<0.0001).^3^

In response to this challenge there is growing interest in employing coatings, with current iterations specifically designed to emulate properties of the endothelial surface.^4^,^7^

Recently, biohybrid hydrogel coatings based on the biopolymer heparin and a synthetic four-armed poly(ethylene glycol) (PEG) with coagulation-responsive linker peptides have been developed that degrade and release the anticoagulant heparin by a local coagulation trigger. Activated coagulation factors thrombin (FIIa) or Factor Xa (FXa) selectively cleave the peptide-linker and release heparin.^8 9^ The hydrogel has been applied successfully on coronary stents.^10^

However, coronary and neurointerventional stents have completely different purposes and designs: Coronary stents are fabricated to treat stenotic diseases and thus are often balloon-expandable devices made of stainless steel or cobalt-chromium with high radial force and struts of >100 µm.^11^ Neurointerventional stents are, on the other hand, mostly self-expandable, with thin struts of <60 µm and are mostly made of nickel-titanium alloy and only rarely of cobalt-chromium. These devices primarily focus on achieving smooth deployment and optimal wall apposition, with less emphasis on radial force.^12^ Moreover, various neurointerventional devices, such as recently developed aneurysm neck-bridging devices, have only temporary applications lasting from minutes to hours before they are removed from the vascular system. This makes them ideal candidates for utilizing a hydrogel technology with limited capacity. In this study, we have chosen stent retrievers (SRs) as a representative surrogate for typical self-expandable, nickel-titanium neurointerventional devices with fine struts because they combine these attributes with the convenience of a facilitated spray coating process, as they can be fully un- and re-sheated by the attached wire. Unlike coronary stents, which are attached to balloons, applying coatings to self-expanding nickel-titanium (NiTi-) based devices poses significant challenges due to the intense friction and shear experienced at the catheter wall during deployment and retrieval.

This study aims to compare bare metal stents (BMS) with devices coated with thrombin-responsive (thrombin-cleavable PEG-heparin gel [tcPHG]) and non-responsive hydrogels (PEG-heparin gel [PHG]) using a Chandler-Loop assay and an animal model. The focus of this study is particularly on the stability and hemocompatibility of the hydrogel under these distinctive conditions.

## Methods

### Stent coating

Stent retrievers of nickel-titanium (pRESET thrombectomy device, 6 mm x 30 mm, Phenox, Bochum, Germany) were cleaned with hypochlorite solution, water, and ethanol for 15 minutes each under ultrasound treatment and then exposed to a low-pressure air plasma for 5 minutes (PlasmaCleaner, Harrick Plasma, Ithaca, NY, USA). BMS were used after this cleaning process without any further treatment.

The hydrogel coating was performed as described previously with a dual silane base layer of 1,2 Bis-(triethoxysilyl)ethane and (3-aminopropyl)trimethoxysilane and a hydrophilic poly(ethylene-alt-maleic anhydride) bonding layer.^10^

Precursor-solutions for tcPHG and PHG were prepared as described previously.^9 10^ Briefly, carboxylic acid groups of heparin (MW 14 kDa, Merck, Darmstadt) were activated using carbodiimide (EDC) and N-hydroxysulfosuccinimide (sulfo-NHS) on ice for 20 minutes and mixed with a 1.5 molar excess of N-terminated four-armed poly(ethylene glycol) (starPEG) (PHG) as the PHG precursor solution. For the tcPHG precursor solution, the activated heparin was reacted with an equimolar amount of starPEG conjugated with the thrombin-cleavable linker-peptide NH_2_-Gly-Gly-(D)Phe-Pip-Arg-Ser-Trp-Gly-Cys-Gly-CONH_2_. The solid content of the precursor solutions was set to 6.7%. For analytical purposes, the heparin in both precursor solutions was spiked with 0.5% Atto647 fluorescently labeled heparin.

The hydrogel coating was prepared by spray-application of the hydrogel precursor solutions on the stents using a standard air-brush device, and the gels polymerized in a humidified atmosphere over night. The hydrogels were swollen in phosphate-buffered saline for 12 hours, and the medium was replaced repeatedly to remove non-polymerized components. The coating success and homogeneity were verified by fluorescent scanning (FLA5100, Fujifilm, Tokyo, Japan) of the stents with a 635 nm excitation laser and a 665 nm longpass emission filter. The hydrogel coatings were dehydrated in increasing ethanol concentrations and air-dried. Glass coverslips were coated as witness samples in parallel to the stents to test the stability and responsiveness of the coating. These samples, after dehydration and air-drying, were rehydrated in 100 mM NaCl, 50 mM Tris(hydroxymethyl) aminomethane (Tris), 0.1% bovine serum albumin (BSA), pH 7.4 for 1 hour. Then, this buffer was replaced with fresh buffer containing 50 nM thrombin for 1 hour, and the hydrogel degradation was determined via the release of the fluorescently labeled heparin.

### Chandler-Loop incubation

Blood was obtained without tourniquet by cubital venous puncture with a 19 G cannula and immediately anticoagulated with 1 U/mL heparin (ratiopharm, Ulm, Germany). The blood of two ABO-compatible donors was pooled to reduce inter-donor variation and used immediately for the experiment. The donors confirmed feeling healthy, and that they had not taken any medication in the past 10 days; their blood cell count and C-reactive protein were in the normal range.

The BMS, PHG and tcPHG coated stents without the delivery wire were inserted separately into ultrasonically cleaned Silicon Tygon tubes (Saint-Gobain, La Défense, France), with a 3.2 mm inside diameter and a 55 cm length. A tube without a stent served as a reference. These tubes were closed to loops, using an external connecting tube and avoiding interfacial steps. The tubes were filled with 3 mL blood and incubated for 1 hour at 37°C, 5% CO_2_. The loops were rotated at 13 rotations per minute, leading to a flow speed of 12 cm/s, mimicking the in vitro vessel geometry and blood flow conditions at the stent (figure 2A).

After the incubation, the stents were harvested, rinsed with PBS and fixed in 2% glutaraldehyde. The stents were cut into two parts along the axis. One part was dehydrated in increasing ethanol concentrations, air-dried, gold-sputtered and inspected by scanning electron microscopy (ESEM Quattro S, Thermo Scientific). The other half was stained with the DNA dye Sybr Green to identify nuclei of adherent cells and analyzed on a fluorescence scanner (FLA5100). The fluorescence intensity on the struts was quantified by ImageJ,^13^ applying masks above a threshold value.

A blood cell count was performed using a Coulter counter (AcT Diff, Coulter, Krefeld, Germany). Blood platelet activation was determined by flow cytometry after blood fixation with ThromboFix (Beckman Coulter, Krefeld, Germany) and staining with CD41a-FITC (Decton-Dickinson (BD), Heidelberg, Germany) for platelet identification and CD62P-PE (BD) as activation marker. The rate of CD62P-positive platelets was quantified. Granulocyte activation was determined by flow cytometry as the expression level of the marker CD11b (PacificBlue conjugate, BioLegend, San Diego, CA, USA) on granulocytes, identified by their forward-/side-scatter behavior and CD15 positivity. Blood was further stabilized with ethylenediaminetetraacetic acid (EDTA) or citrate, theophylline, adenosine, and dipyridamole (CTAD), as recommended by the test kits, spun down, and plasma was analyzed by ELISA for the prothrombin fragment F1+2 (Enzygnost F1+2, Siemens Healthineers, Erlangen, Germany), Platelet factor 4 (PF4, Zymotest PF4, CoaChrom, Vienna, Austria) and the complement fragment C5a (C5a micro, DRG, Marburg, Germany).

### Animal experiments

All experiments were conducted in compliance with the ARRIVE guidelines. This study used five female New Zealand white rabbits (Charles River, Sulzfeld), aged 22 weeks. All procedures were performed under general anesthesia, administering intramuscular ketamine at 60 mg/kg (Ketamidor, WDT, Germany) and xylazine at 6 mg/kg (Serumwerk Bernburg AG, Germany). Anesthesia was maintained using the same amount of ketamine (60 mg/kg) and xylazine (6 mg/kg) in 10 mL NaCl 0.9%, administered at a flow rate of 2.5 mL/h via an ear vein.

The left femoral artery was surgically exposed, and a 4 French sheath (Terumo, Tokyo, Japan) was inserted. A 0.021-inch microcatheter (Trevo Trak, Stryker, Kalamazoo, USA) was navigated over various 0.014 inch microwires to access the subclavian and common carotid arteries for stent deployment.

Each of the three types of stents (BMS, PHG, tcPHG) was randomly deployed in every animal. No anticoagulation or antiplatelet was administered to the rabbits. After an hour of incubation, euthanasia was induced by a pentobarbital overdose (Narkodorm, cp-pharma, Burgdorf, Germany). After removing the wires, the harvested stents underwent the same analysis conducted on the stents from the Chandler-Loop incubation.

### Statistical assessment

Data of Chandler-Loop incubations are presented as mean and standard deviation (SD) of four sets of stents analyzed in two independent incubation settings. Rabbit in vivo experiments were performed with three sets of samples in five rabbits.

## Results

### Properties of the hydrogel coating

**Figure 1:**
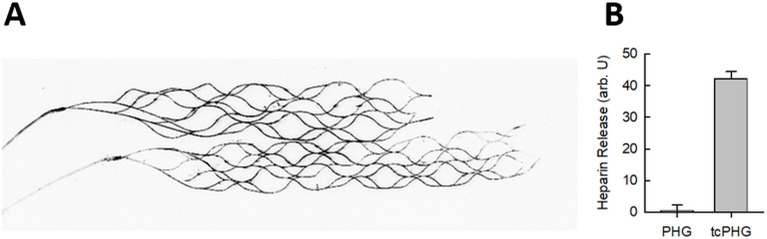
Properties of the hydrogel coating. (**A**) Fluorescent scan of the stent retrievers with the fluorescently labeled hydrogel coating. No coating was applied to the delivery wires of the stent, which, therefore, appear pale and confirm the successful coating application on the stent. (**B**) Hydrogel degradation in thrombin solution. The non-responsive PHG sample shows virtually no release of fluorescently conjugated heparin compared with the high release from the thrombin-cleavable tcPHG sample.

Fluorescent scans showed the homogeneous distribution of the gels on the stent bodies (figure 1A). The tcPHG with the thrombin-cleavable linker peptide released heparin when incubated with thrombin, whereas there was no release from the directly crosslinked PHG hydrogel, confirming that both coatings did exhibit their intended claimed activity (figure 1B).

### Chandler-Loop

**Figure 2:**
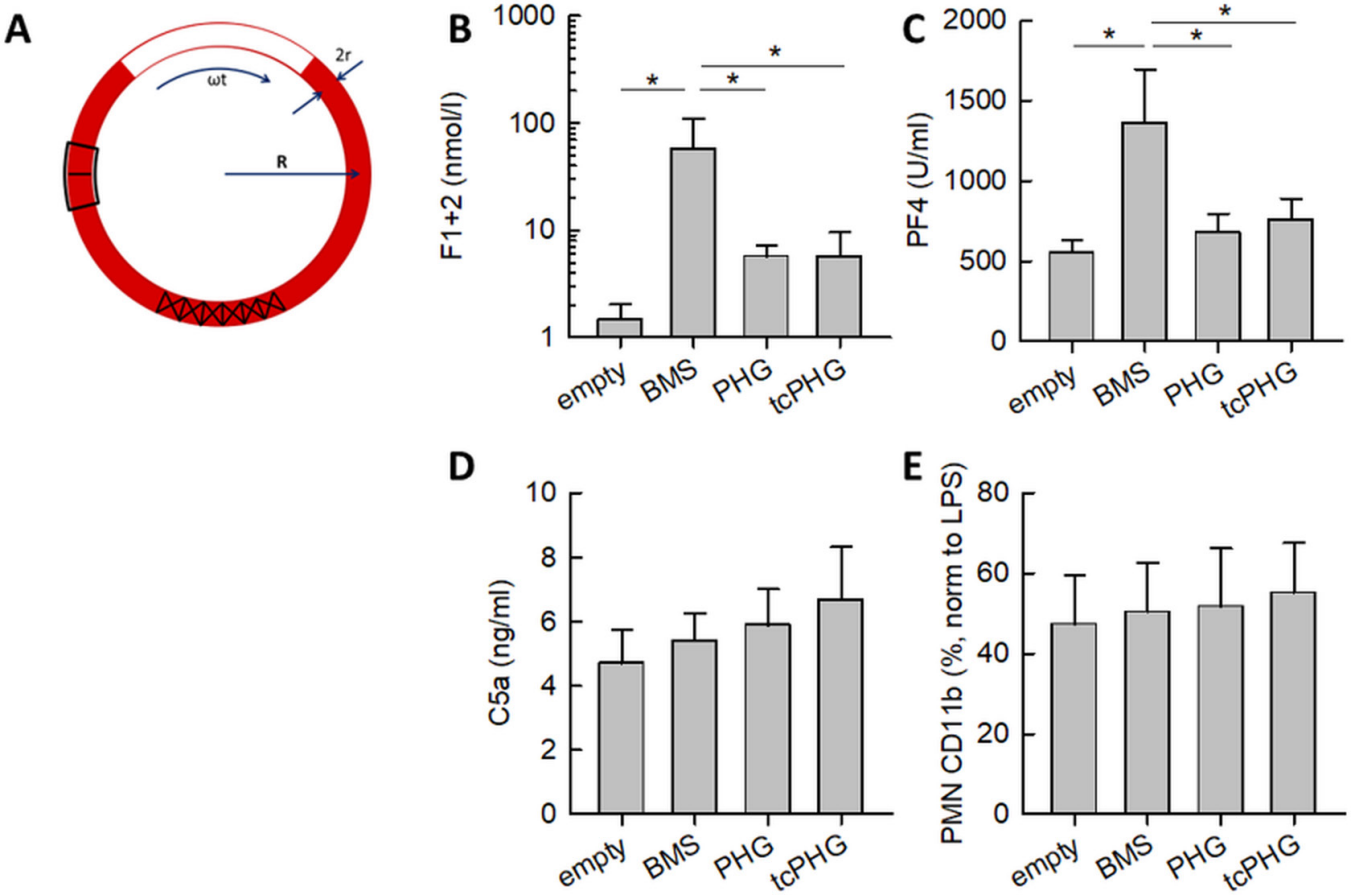
Whole blood response to bare metal stent retrievers (BMS), stent retrievers with starPEG-Heparin hydrogel (PHG) or thrombin-cleavable starPEG-Heparin hydrogel (tcPHG) coating during 1 hour Chandler-Loop incubation compared with an empty Chandler-Loop without stent. (**A**) Principle of the Chandler-Loop: the stent is inserted in a silicon tube that is partly filled with blood. Rotation of the tube causes a blood flow due to gravity. (**B**) Activation of the coagulation cascade measured as the prothrombin fragment F1+2. (**C**) Platelet activation, measured as the release of platelet factor 4 (PF4). (**D**) Complement activation measured as the formation of the complement fragment C5a. (**E**) Granulocyte activation measured as exposure of the activation marker CD11b. Data are normalized to the response to 100 U/mL endotoxin.

The BMS induced a significant increase in the activation of the coagulation cascade and blood platelets compared with the empty tube. This was measured as prothrombin F1+2 fragment and platelet factor 4 (PF4) release, respectively (figure 2B,C). The hydrogel coatings on the stents reduced the thrombin activation to about 10% of the level of the BMS, but not to the baseline level. This reduction remained consistent regardless of whether the hydrogel was thrombin-cleavable (tcPHG) or not (PHG). Hydrogel coating reduced the PF4 release from platelets almost to the baseline level, independent of the type of hydrogel.

The humoral and cellular inflammatory response to the coated stents, measured as complement C5a formation and as granulocyte CD11b expression, were only marginally elevated compared with the empty tubes or BMS (figure 2D,E).

**Figure 3:**
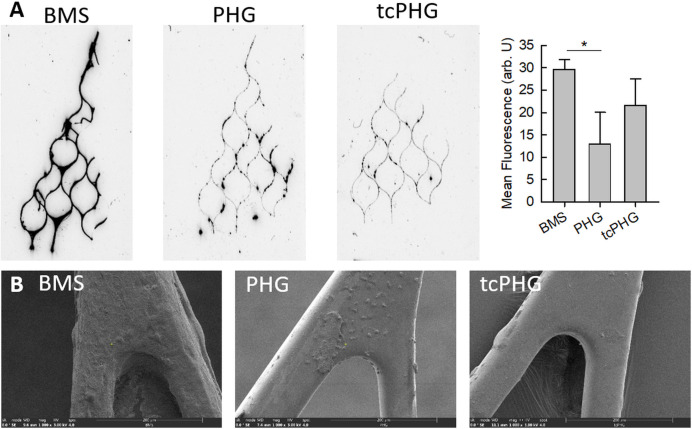
Stent retrievers after 1 hour of Chandler-Loop incubation. (**A**) Fluorescent scan of SybrGreen stained stent fragment and quantitative evaluation. (**B**) Corresponding scanning electron microscopy images.

After incubation, the stents were tested for adherent blood clots using fluorescent dyes that label clot components. Figure 3A shows fluorescent scans of the stents with different coatings. There was obvious clot formation at the struts of the BMS stent, with a preference on the branchings. Substantially less clotting was observed at the hydrogel-coated stents, with the PHG showing a tendency to outperform the tcPHG.

The gross results of the fluorescent scanner are supplemented by scanning electron microscopy images (figure 3B). The struts of the bare metal stent were covered with a fibrin mesh containing entrapped blood cells. Only a few granulocytes and platelets adhered on both types of hydrogel-coated stents.

### Animal **e**xperiments

**Figure 4:**
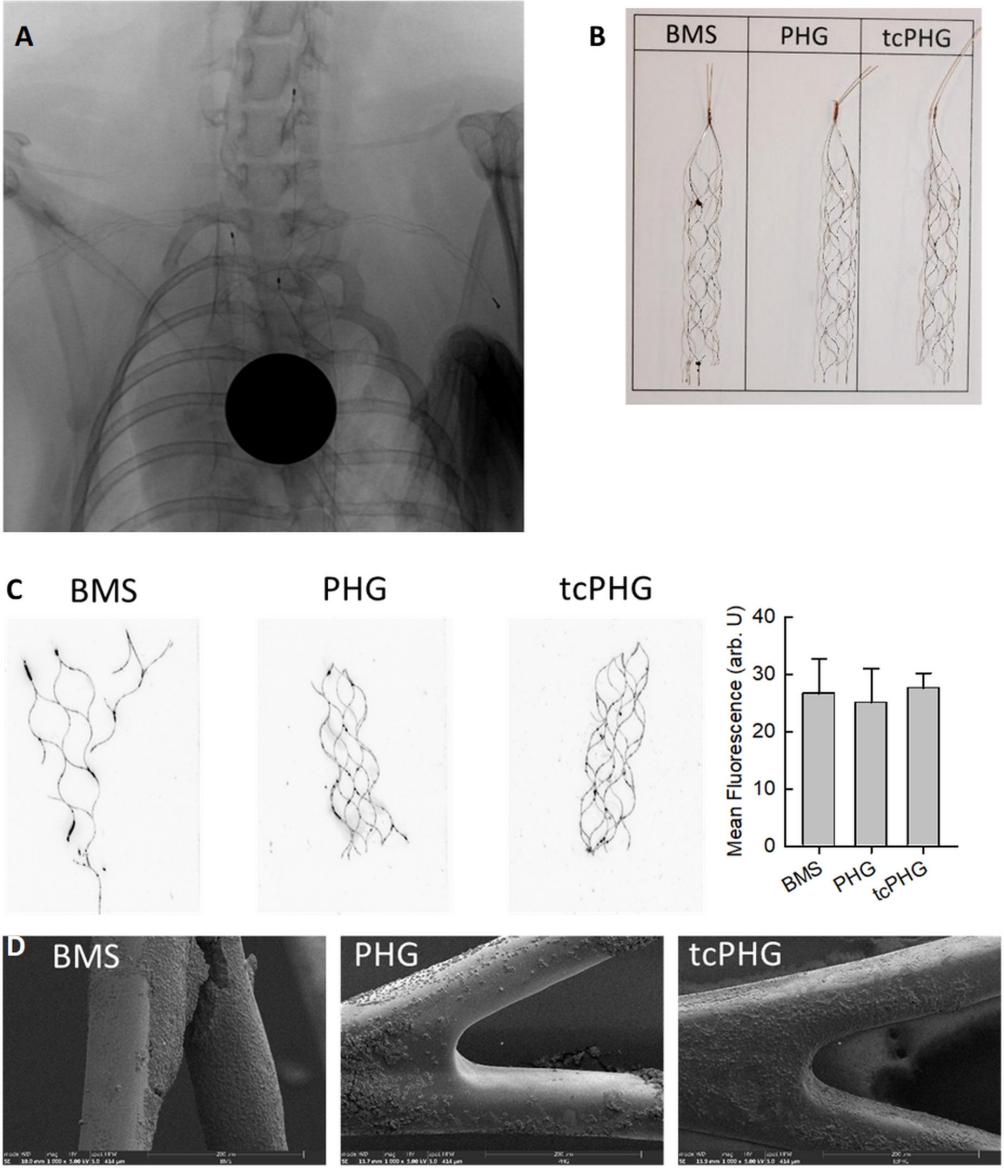
Stent retrievers after 1 hour of implantation in rabbit subclavian and carotid arteries. (**A**) X-Ray of the stents in the rabbit’s supra-aortic vessels. (**B**) Photographs of the stents. (**C**) Scans of fluorescent DiOC6 stents and quantitative evaluation (**D**) SEM images.

After 1 hour of implantation into rabbit carotid and subclavian arteries (figure 4A), the stents were harvested by retraction through the sheath and subjected to the same analysis as those from Chandler-Loop incubation. Visual inspection showed random stent-associated clots up to ca. 1 mm diameter, predominantly at the branchings of the stents (figure 4B). There was no overall quantitative difference in clot deposits between the BMS and hydrogel-coated stents (figure 4C), although SEM images suggested a relatively dense clot deposition at BMS, whereas only individual cells adhered at the hydrogels PHG and tcPHG (figure 4D).

## Discussion

Several neurovascular interventional devices have limited blood exposure time in standard application. They are usually not equipped with an antithrombotic coating for the short contact time. However, this study shows that the 1 hour handling time of catheters and wires in circulation can lead to the deposition of thrombi. Such device-associated thrombosis can be reduced with systemic antiplatelet therapy or anticoagulation of the patient.^4 14^ However, this treatment is associated with increased bleeding risk and consequently, less aggressive antithrombotic therapies are desirable^3 15^; although peri- and post-procedural thromboembolic complications still pose a significant challenge.^1^ Antithrombotic coatings on stents, wires and catheters thus may increase their safety profile.

This study applied recently developed feedback-controlled anticoagulant hydrogels as coatings on neurovascular self-expanding NiTi stents.^9 10^ These thrombin-cleavable starPEG-heparin hydrogels (tcPHG) release the anticoagulant heparin in response to the activated coagulation factor thrombin and thus inhibit the blood coagulation in a situation-adjusted manner. Non-responsive starPEG-heparin hydrogel coatings (PHG) and the bare NiTi alloy here served as references. This coating technology is compatible with standardized industrial processes, but it was performed here on a less standardized laboratory level. The thickness and homogeneity of the coating were not controlled. Water evaporation during the spray process alters the actual solid content during the polymerization to a non-controlled level. Witness samples, therefore, were prepared to evaluate the stability and responsiveness of the final coating. Additionally, we tested the distribution of the coating on the stents using a fluorescence scanner, confirming its successful application. Our results also confirm the coating’s stability after deployment through the catheter, despite the intense shear stress exerted on the densely folded stents.

The Chandler-Loop incubation allowed the use of fresh human blood, which is closest to the target application. It is a constant pool system where activation and release products accumulate in the blood volume during incubation and may reach higher concentrations than in the body, facilitating the analysis of these processes. The flow velocity of 12 cm/s in this study is representative of the intracranial venous system.^16^

The New Zealand rabbit is widely accepted as the standard model for preclinical evaluation of neurointerventional devices.^17 18^ The flow velocities in the supra-aortic arteries (10–50 cm/s, diastolic and systolic, respectively)^19^ are closer to the values of human intracranial arteries. In contrast to the Chandler-Loop, there is no constant pool situation, and in addition, blood vessel cells can exhibit their modulating effect on the blood coagulation. However, the coagulation system in rabbit blood slightly differs from human blood. Rapid clotting with kaolin but low sensitivity to other activated partial thromboplastin time (aPTT) reagents has been reported,^20 21^ suggesting differences in the contact phase activation compared with human blood. Reduced tissue factor-dependent thrombin formation has been described for rabbits,^21^ whereas the platelet reactivity of rabbits appears similar to humans.^22^ While such species-specific differences have to be considered in a test model, their influence on the results is hard to quantify.

The in vitro Chandler-Loop assay allowed separate blood incubation of the individual stents and consequently individual analysis of activation processes in blood by the stents. In contrast, each rabbit got the three types of stents implanted in parallel to avoid interindividual differences. Analysis of the rabbit blood would not be informative in evaluating stent properties, and only the stents were analyzed.

In this study, scanning electron microscope (SEM) images both after the Chandler-Loop and the rabbit model showed clot formation at the branchings of the struts of the BMS and only minor deposits on both types of hydrogel coatings. The clots in the Chandler-Loop model mainly consisted of fibrin fibrils and platelets, whereas in the rabbit model, the clots had less fibrin but more cells. This difference may result from the predominance of humoral coagulation induction in the venous system, which was reproduced in the Chandler-Loop. In contrast, platelet-induced clot formation predominates in the arterial high-shear system, as reproduced in the rabbit model.^23^ This highlights the different requirements for hemocompatible coatings in low- and high-shear applications.^24^ The coagulation cascade predominates over platelet activation in clot formation under static and venous flow conditions. Via contact phase activation and the intrinsic pathway of the coagulation cascade, plasmatic coagulation also dominates over platelet aggregation in device-associated thrombosis. It can be inhibited by anticoagulants like the released heparin. Under arterial high-shear conditions, however, platelet activation and adhesion to von Willebrand factor predominate, requiring different drugs.^25^ In the in vivo system, also mechanically or chemically activated endothelium may contribute to the observed clot formation, which was not analyzed in this study.

These findings hold significant relevance for the neurointerventional field, particularly in the realm of stent coatings aimed at enhancing hemocompatibility. Companies such as Medtronic and Phenox have been at the forefront, with Medtronic’s Shield utilizing a phosphorylcholine polymer and Phenox’s glycan-based hydrophilic polymer coating (HPC) mimicking the vessel wall’s glycocalyx.^4 5^ Clinical data support the safety of these coatings under single-antiplatelet therapy (SAPT), with ongoing trials further investigating their efficacy.^26^ Additionally, companies such as MicroVention and Stryker are actively pursuing innovative coating technologies, such as a biopassive poly-2-methoxyethyl acrylate (PMEA) or bioactive heparin coating, to improve hemocompatibility and endothelialization.^7 27^ More technologies are currently under investigation, aiming at improved hemocompatibility and/or endothelialization. Importantly, recent data indicate that current coating technologies do not exacerbate complications such as non-ischemic cerebral enhancing (NICE) lesions associated with neuroendovascular procedures.^28^

The tcPHG coating investigated in this study derives its antithrombogenic properties from two mechanisms of action: (1) the bioactive heparin release triggered by activation of the coagulation cascade, which is an additional feature to the above-cited stable heparin coating by Stryker/Carmeda (Solna, Sweden); and (2) the formation of a protective boundary layer of intermediate water,^29^ which is thought to be even stronger than with PMEA coatings. This explains why PHG itself has pronounced antithrombogenic properties and was superior to BMS in all experiments, which is a potentially useful observation.

The heparin release from the tcPHG did not exhibit an additional benefit over the stable PHG coating, although we demonstrated a relevant heparin release in our experiments. Potentially, an additional positive effect of the bioactive component in tcPHG may become evident by longer incubation times in vitro and in vivo or by permanent implantation of the device with long-term follow-up.

This type of data will also be necessary to examine the potential effects of tcPHG/PHG on the device’s endothelialization, as starPEG may impair cell adhesion in a more pronounced fashion than, for example, PMEA.^7^ Yet, even without these data tcPHG/PHG is already a potentially interesting candidate to increase the hemocompatibility of venous stents or temporarily introduced systems and devices, such as catheters, remodeling balloons or temporary neck-bridging devices, a novel device class consisting of self-expandable nickel-titanium mesh structures that are used to assist the coil embolization of intracranial aneurysms.^30 31^

## Conclusion

Altogether, this study demonstrates the beneficial effect on hemocompatibility of stable and coagulation-responsive starPEG-heparin hydrogels (PHG and tcPHG, respectively) both in vitro and in vivo, although no significant difference was confirmed between both PHG-based coatings. The advantage of tcPHG and PHG over BMS was more pronounced in vitro, what may be attributed to the higher shear-induced platelet activation in the arterial placement of the stent retrievers, highlighting the different requirements of blood-contacting devices in low- and high-shear conditions representing venous and arterial stent applications. Moreover, our results confirm the structural integrity of the hydrogel after pushing the stent retrievers through the tight lumen of a microcatheter. Different blood exposure times might better elaborate a difference between the hydrogel types.

## Data Availability

Data are available in a public, open access repository.
